# Low Back Pain and Swelling as an Atypical Presentation of IgA Vasculitis

**DOI:** 10.5811/cpcem.2019.11.44574

**Published:** 2020-04-14

**Authors:** Clay T. Winkler, Raymond W. Dobson, Michael J. Tranovich

**Affiliations:** * Prisma Health - University of South Carolina School of Medicine, Department of Emergency Medicine, Columbia, South Carolina; † West Virginia School of Osteopathic Medicine, Lewisburg, West Virginia; ‡ Ohio Valley Medical Center, Department of Emergency Medicine, Wheeling, West Virginia

**Keywords:** IgA vasculitis, Henoch-Schonlein purpura, HSP, lumbar swelling

## Abstract

**Introduction:**

Immunoglobulin A vasculitis (IgA vasculitis), formerly Henoch-Schonlein purpura, is the most common vasculitis in children.

**Case Report:**

A 6-year-old female presented with low back pain and swelling, difficulty ambulating, and rash two weeks after a respiratory infection. She was approached with a broad differential and ultimately diagnosed with IgA vasculitis.

**Discussion:**

Cutaneous manifestations, arthralgias, renal and gastrointestinal involvement are the most common presenting signs of IgA vasculitis. Only two cases of IgA vasculitis associated with lumbar pain and swelling were identified in the literature.

**Conclusion:**

While rash and joint pain are common presenting signs of IgA vasculitis, practitioners should be aware it can present atypically.

## INTRODUCTION

Immunoglobulin A (IgA) vasculitis is the most common vasculitis in children.^1^ IgA vasculitis, formerly known as Henoch-Schönlein purpura, is comprised of a clinical tetrad of palpable purpura, arthralgias, abdominal pain, and renal disease, all in the absence of thrombocytopenia or coagulopathy.^1^ Subcutaneous edema, either periorbital or dependent edema in the hands, feet, and genitals, are all well documented in the literature.^1^–^3^ The diagnosis of IgA vasculitis is typically made by clinical examination and presence of the palpable purpuric rash along with two to three of the other classical findings of the clinical tetrad, although IgA deposition can aid the diagnosis.^1^ We present a case of a six-year-old female who presented to the emergency department (ED) due to non-traumatic back pain, swelling, and refusal to ambulate.

## CASE REPORT

A six-year-old female was brought by a family member to the ED complaining of low back pain and swelling. The patient’s symptoms had been ongoing for several days. She denied any history of trauma. She was refusing to ambulate secondary to pain in the joints of the lower extremities. She also noted mild nausea, ankle swelling, and a rash on her lower extremities bilaterally. The patient’s family also described her as having a previous upper respiratory infection approximately two weeks prior. The patient was fully immunized and had an unremarkable past medical, surgical, and family history. She had been taking ibuprofen for her symptoms, but otherwise took no medications.

**Image 1:**
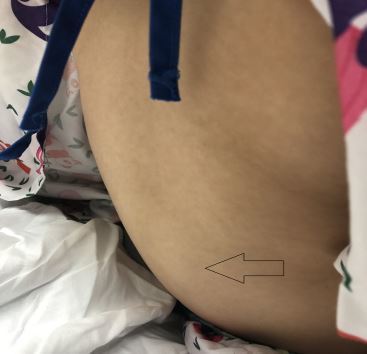
Photograph of the patient’s lower back demonstrating lumbar soft tissue swelling indicated by the arrow.

She was afebrile with a temperature of 99.0° Fahrenheit, and had a heart rate of 112 beats a minute, a blood pressure of 123/81 milligrams of mercury, a respiratory rate of 18 breaths per minute, and 99% pulse oximetry on room air. She was refusing to ambulate secondary to pain but was otherwise nontoxic appearing. There was an oval area of cutaneous edema, similar in shape to an American football, approximately 10 centimeters (cm) vertically and 8 cm transversely, stretching from her lower thoracic spine down to her lower lumbar spine over which she had moderate tenderness (Image 1). She had mild, non-pitting pedal edema and was noted to have a palpable purpuric rash over her ankles and lower legs. Abdominal and neurologic examinations were benign, and the remainder of the physical exam was unremarkable.

**Image 2:**
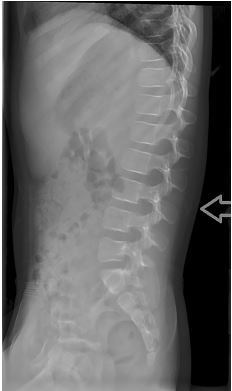
Lateral radiograph of the lumbar spine with noted subcutaneous edema and soft tissue swelling noted by the arrow.

Laboratory studies included an unremarkable complete blood count, prothrombin time, partial thromboplastin time, and complete metabolic panel. Urinalysis was significant for hematuria with 10–15 red blood cells seen per high-powered field on microscopy and without proteinuria. The patient was also noted to have an elevated erythrocyte sedimentation rate at 44 millimeters per hour (normal is <30) and an elevated C-reactive protein at 1.8 milligrams per liter (normal is <1.0). Radiographs of the lumbar spine demonstrated subcutaneous edema but were otherwise unremarkable (Image 2).

The patient was treated with a weight-based dose of ibuprofen and was observed ambulating without difficulty around the ED. She was discharged with a 48-hour follow-up in the ED. At her follow-up it was noted that she no longer had any pain or swelling of her lower back and the remainder of her symptoms were resolving as well.

**CPC-EM Capsule**

What do we already know about this clinical entity?

Immunoglobulin A (IgA) vasculitis is a common pediatric disease that is a combination of a rash, arthralgias, abdominal pain, and renal disease with varying penetrance.

What makes this presentation of disease reportable?

This presentation of IgA vasculitis is a rare presentation only previously described in the literature twice.

What is the major learning point?

The major learning point is that IgA vasculitis will commonly have atypical presentations and should be considered in unusual cases.

How might this improve emergency medicine practice?

This article helps highlight the importance to consider IgA vasculitis in the differential while treating the pediatric patient in the emergency department.

## DISCUSSION

IgA vasculitis is known to have varying presentations, sometimes including edema of the hands, feet, and even genitals.^2^,^3^,^6^ Varying penetrance of clinical features appears to be somewhat common and is well documented.^2^,^6^ In one retrospective study of 150 patients diagnosed with IgA vasculitis, all had cutaneous manifestations, whereas 74% had arthralgias, 54% had renal involvement, and 51% had abdominal involvement.^2^ Gastrointestinal symptoms occur in approximately 50% of cases and can range from nausea and vomiting to intestinal bleeding or intussusception.^1^ Renal involvement can range from a small amount of hematuria or proteinuria to nephrotic-range proteinuria and elevated creatinine.^1^ Diagnosis is usually clinical and based on the presence of classical symptoms, but can be aided by skin and renal biopsy demonstrating IgA deposits.^1^ Complications of IgA vasculitis include chronic kidney disease or, rarely, intussusception.^2^ Treatment is generally aimed at symptom relief; use of corticosteroids is controversial.^1^,^2^

After a literature review, we identified only two previous cases of IgA vasculitis associated with lumbar pain and swelling in a child diagnosed with IgA vasculitis.^4^,^5^ In one of these cases, the patient’s presenting symptoms were abdominal pain rather than back pain. This patient had hemoccult-positive stool and only later developed the lumbar swelling on day 3 of hospitalization.^4^ The lumbar swelling resolved after two days; however, that patient did develop facial edema that required ongoing corticosteroid treatment.^4^ In the second case, the patient’s presenting symptom was back pain and refusal to ambulate after a febrile illness and development of a rash. Magnetic resonance imaging was performed, which demonstrated edema of the lumbosacral fascial planes.^5^

The lumbar swelling noted in those two cases and in our case is likely the same mechanism of dependent edema, which commonly leads to swelling of the hands and feet. We approached our patient with a broad differential diagnosis. After a thorough physical examination and laboratory studies, she was diagnosed with IgA vasculitis, treated accordingly, and discharged home.

## CONCLUSION

This case illuminates an atypical presentation of IgA vasculitis that is notable for its comparative rarity, having been noted only twice previously in the literature.^4^,^5^ The patient’s atraumatic low back pain and swelling elicited a wide differential diagnosis on her presentation. Only after a thorough examination and laboratory studies did we determine that it was a presentation of IgA vasculitis. This case demonstrates the importance for clinicians to be aware that this common diagnosis can present in uncommon ways.
